# Carbohydrate response element binding protein (ChREBP) correlates with colon cancer progression and contributes to cell proliferation

**DOI:** 10.1038/s41598-020-60903-9

**Published:** 2020-03-06

**Authors:** Yu Lei, Shuling Zhou, Qiaoling Hu, Xueling Chen, Jiang Gu

**Affiliations:** 1 Department of Pathology and Provincial Key Laboratory of Infectious Diseases and Immunopathology, Collaborative and Creative Center, Shantou University Medical College, Shantou, 515041 Guangdong China; 2 Jinxin Research Institute for Reproductive Medicine and Genetics, Chengdu Jinjiang Hospital for Maternal and Child Health Care, 66 Jingxiu Road, Chengdu, 610066 China

**Keywords:** Colorectal cancer, Tumour biomarkers, Cell growth

## Abstract

Cancers are characterized by reprogrammed glucose metabolisms to fuel cell growth and proliferation. Carbohydrate response element binding protein (ChREBP) is a glucose-mediated transcription factor that strongly regulates glycolytic and lipogenic pathways. It has been shown to associate with metabolic diseases, such as obesity, diabetes and non-alcoholic fatty liver diseases. However, how it associates with cancers has not been well understood. In this study, ChREBP expression was assessed by immunohistochemistry in colon tissue arrays containing normal colon tissue and cancer tissue at different clinical stages. Tissue mRNA levels of ChREBP were also measured in a cohort of colon cancer patients. We found that ChREBP mRNA and protein expression were significantly increased in colon cancer tissue compared to healthy colon (p < 0.001), and their expression was positively correlated to colon malignancy (for mRNA, p = 0.002; for protein p < 0.001). Expression of lipogenic genes (*ELOVL6* and *SCD1*) in colon cancer was also positively associated with colon malignancy (for both genes, p < 0.001). *In vitro*, ChREBP knockdown with siRNA transfection inhibited cell proliferation and induced cell cycle arrest without changes in apoptosis in colon cancer cell lines (HT29, DLD1 and SW480). Glycolytic and lipogenic pathways were inhibited but the p53 pathway was activated after ChREBP knockdown. Taken together, ChREBP expression is associated with colon malignancy and it might contribute to cell proliferation via promoting anabolic pathways and inhibiting p53. In addition, ChREBP might represent a novel clinical useful biomarker to evaluate the malignancy of colon cancer.

## Introduction

Carbohydrate response element binding protein (ChREBP) is a basic helix–loop–helix leucine zipper (bHLH-LZ) transcription factor that is mainly expressed in liver, white and brown adipose tissue, intestine, muscle, and pancreatic β-cells^1^. It was identified by Uyeda and colleagues in 2001 as the principal mediator of the transcriptional effects of glucose^2^. In response to increased glucose levels, ChREBP undergoes dephosphorylation and translocates from the cytoplasm to the nucleus where, it associates with its functional obligatory partner MLX (Max-like interacting protein), to form a heterodimer. This heterodimer binds to carbohydrate responsive elements (ChoRE) of ChREBP target genes in the nucleus^3,4^. ChREBP can be activated by glucose metabolites (includes Glucose-6-phosphate, Xylulose-5-phosphate, fructose-2.6-bisphosphate)^5–7^, and by post-translational modifications, such as acetylation^8^ and O-GlcNAcylation^9^. The ChREBP/MLX heterodimer controls glucose and lipid metabolism via regulating the expression of glycolytic (*Pklr, Fk, Glut2, Glut4*), gluconeogenic (*G6pc*), and lipogenic (*Fasn, Acc1, Scd1, Elovl6*) genes^1,4,10^, suggesting that ChREBP may have an important role in the pathogenesis of metabolic diseases and cancer. So far, most of the studies of ChREBP originated from mouse models and have focused on its function as a hepatic transcription factor to regulate glucose and lipid metabolism. Not much is known about the role of ChREBP in human, especially in cancer cells. The relationship of ChREBP and cancer malignancy and how ChREBP influences cell proliferation, apoptosis and cell cycle have not been well investigated.

More and more studies have shown that perturbed cellular metabolism is associated with tumor development^11,12^. Metabolic diseases such as obesity and diabetes are associated with increased risk to develop cancer including breast cancer, colon cancer, prostate cancer and pancreatic cancer^13–15^. In addition, many human tumors display a high rate of aerobic glycolysis, *de novo* fatty acid synthesis and nucleotide biosynthesis^16,17^. It has been proposed that increased glucose metabolism promotes lipogenesis and nucleotide biosynthesis, and enhances tumor cell growth and proliferation by providing essential energy and building blocks for cell proliferation^18,19^. Increased *de novo* fatty acid synthesis is required in cancer cells for the construction of lipid membranes, and inhibition of fatty acid synthetase FAS inhibited cell proliferation and increased apoptosis of tumor cells^20^. Since ChREBP strongly regulates glucose and lipid metabolism, it would be logical to investigate ChREBP expression in human cancer.

It has been reported that the level of ChREBP protein expression is positively correlated with tumor progression in breast cancer^21^. Recently, ChREBP was also reported to be associated with hepatocellular carcinoma progression^22–24^. However, ChREBP expression has not been examined in human colon cancer and the relationship between ChREBP expression and the degree of colon tumor malignancy has not been reported. Colorectal cancer (CRC) is one of the most deadly tumors worldwide. In addition to genetic modifications, factors causing metabolic alterations such as obesity, sedentary life style and Westernized diet are risk factors for this disease^25^.

It has been reported that ChREBP can direct glucose metabolism from oxidative phosphorylation to anabolic pathways and is required for the proliferation of various cell types^21,26–28^. ChREBP suppression in HCT116 cells and HepG2 hepatoblastoma cells decreases aerobic glycolysis and anabolism, and is accompanied by decreased synthesis of lipids and nucleotides^26^. Suppression of ChREBP reduces glucose-induced pancreatic β-cell proliferation as well as mRNA levels of cell cycle regulators while conversely overexpression of ChREBP promotes glucose-stimulated β-cell proliferation^27,29^. These findings suggest an essential role of ChREBP in regulating cell proliferation and cell cycle.

In this study, we analyzed ChREBP protein expression in a human colon tissue array composed of normal and cancer tissue at different clinical stages. mRNA expression of ChREBP and its lipogenic target genes were analyzed in another cohort of colon cancer patients. We found that the expression of ChREBP mRNA and protein were significantly higher in cancer cells in comparison to normal colon, and their expression were positively associated with advanced stages of cancer. *In vitro*, experiments were performed in 4 colon cancer cell lines, including 3 p53-positive cell lines (HT29, DLD1, SW480) and 1 p53 function disrupted cell line (RKO-E6). ChREBP knockdown mediated by siRNA inhibited cell proliferation in all 4 cell lines and induced cell cycle arrest in HT29 and DLD1 cells. ChREBP knockdown did not influence cell apoptosis. Glycolytic and lipogenic pathways were inhibited but the p53 pathway was activated by ChREBP knockdown. Taken together, this study demonstrates that ChREBP is required for cell proliferation and ChREBP expression positively associated with colon cancer progression and might serve as a marker in evaluation of colon cancer behavior.

## Results

### ChREBP mRNA and protein levels positively correlate to colon malignancy

**Figure 1:**
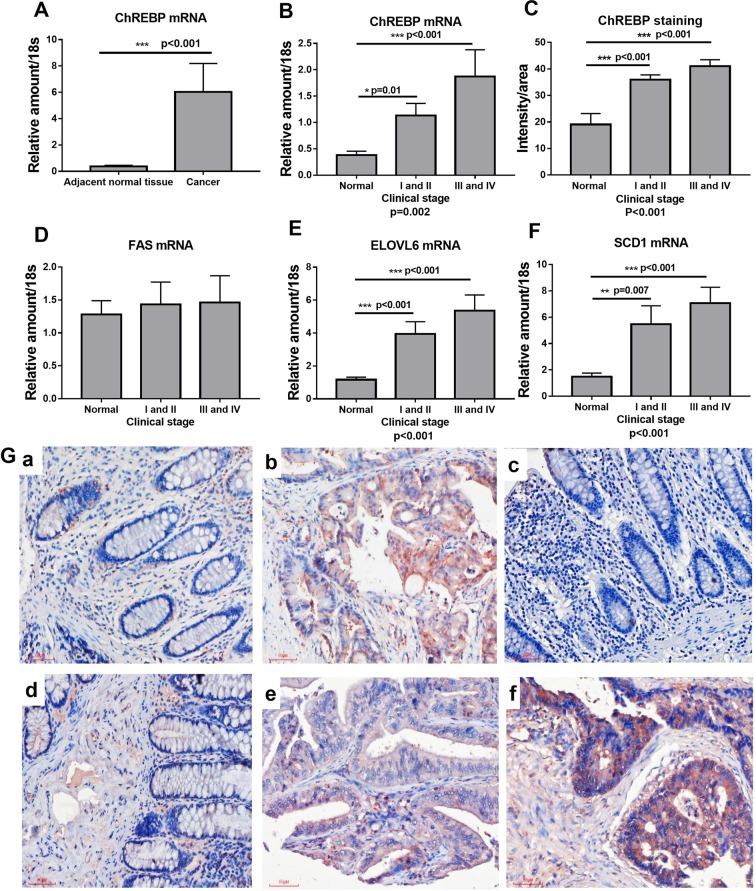
ChREBP mRNA and protein levels positively correlate to colon malignancy. (**A**) Relative mRNA level of ChREBP normalized to 18s in 20 pairs of colon cancer tissue and adjacent normal tissue. (**B**) Relative mRNA level of ChREBP normalized to 18s in normal, early and late clinically staged colon cancer tissue. (C) ChREBP protein expression in normal, early and late clinically staged colon cancer tissues. Relative mRNA levels of FAS (**D**), ELOVL6 (**E**) and SCD1 (**F**) normalized to 18s in early and late clinically staged colon cancer tissue. (**G**) ChREBP immunohistochemistry in healthy and malignant colon sections. The strong ChREBP positive staining in malignant colon (b) compared with an mild staining in adjacent normal colon tissue (a) in the same patient; c, a negative immunohistochemical staining control, which was incubated with PBS instead of preliminary antibody; d, healthy colon tissue; e, clinical stages I and II colon cancer tissue; f, clinical stages III and IV colon cancer tissue. Photos were taken under the magnification of 200×. Scale bar = 30 μm. *p < 0.05; **p < 0.01; ***p < 0.001.

To investigate whether there is an association between ChREBP expression and colon malignancy, qPCR and immunohistochemistry were performed on these colon samples. At the mRNA level, ChREBP mRNA increased significantly when compared to the adjacent normal colon tissues (Fig. 1A). It showed more ChREBP expression in malignant cancer cells than in normal colon cells in the same patients. Moreover, ChREBP mRNA levels increased with the stage of colon cancer. The result of one-way ANOVA of ChREBP mRNA level and clinical stage revealed that ChREBP mRNA expression increased significantly (p = 0.002) with colon malignant progression (Fig. 1B). At the protein level, ChREBP immunohistochemical staining results showed that intensity of the signal increased with malignant progression as defined by histopathological diagnosis (Fig. 1C and 1G). The staining positivity was quantified with software Image J. The result of one-way ANOVA of ChREBP expression and clinical stage indicated that ChREBP expression increased with clinical stage (p < 0.001) (Fig. 1C). In the same patient, the staining of ChREBP in cancer issue (Fig. 1Gb) was stronger than that of the adjacent normal colon tissue (Fig. 1Ga). The color of ChREBP staining also changed from light red to dark red with colon cancer progression (Fig. 1Gc–f).To investigate how glycolysis and lipogenesis associate with colon cancer progression, the protein levels of glucose transporter, GLUT1 and GLUT2, involved in glycolysis^30,31^ were measured with immunohistochemistry and semi-quantification. We found that both GLUT1 and GLUT2 were significantly increased in cancer tissues and their expressions were correlated with clinical stage (GLUT1, p < 0.001; GLUT2, p = 0.001) (Fig. S1A–B). The mRNA levels of lipogenic genes, elongation of very long chain fatty acids protein 6 (ELOVL6) and stearoyl-Coenzyme A desaturase-1 (SCD1*)*, which are also ChREBP targets, were significantly increased in colon cancer tissues when compared to normal colon tissue and these increase were positively associated with colon cancer progression (for both genes, p < 0.001), but this trend did not applied to fatty acid synthase (FAS) (Fig. 1D–F).

### ChREBP knockdown inhibited glycolysis and lipogenesis but activated the p53 pathway in colon cancer cells

**Figure 2:**
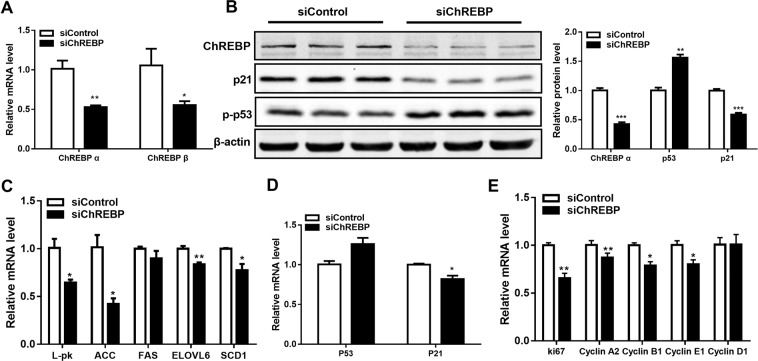
ChREBP knockdown inhibited glycolysis, lipogenesis and p21 in HT29 cells. (**A**) Inhibited relative mRNA expression of ChREBP α and ChREBP β normalized to B2M after control or ChREBP siRNA transfection for 48 hours. (**B**) Western-blot of ChREBP, phospho-p53 and p21 and their quantification on the right. β-actin was served as a loading control. Proteins were extracted from cells transfected with sicontrol and siChREBP after 48 hours. The quantification of western blot was normalized to β-actin. (**C**) Decreased relative mRNA expression of glycolytic and lipogenic genes, normalized to B2M. (**D**) Relative mRNA expression of p53 and p21, normalized to B2M. (**E**) Relative mRNA expression of cell Cyclins, normalized to B2M. n = 4. *p < 0.05; **p < 0.01; ***p < 0.001.

To investigate the role of ChREBP in cell proliferation, cell cycle and apoptosis *in vitro*, ChREBP was knocked down with siRNA in colon cancer cell lines. In HT29 cells, with the siChREBP transfection, ChREBP α and ChREBP β mRNA levels were decreased by 47% and 45%, respectively, when compared to cells treated with siControl (Fig. 2A). As shown by Western blot, ChREBP protein level was decreased by 57% when compared to the control group (Fig. 2B). These mRNA and protein data indicated that ChREBP had been sufficiently inhibited in HT29 cell. Gene expression of liver pyruvate kinase (L-pk), which is an essential gycolytic gene, was significantly inhibited by 36% following ChREBP knockdown. Genes involved in the lipogenic pathway, including acetyl-CoA carboxylase (ACC), ELOVL6 and SCD1 were significantly inhibited by 60%, 16% and 20%, respectively. FAS was slightly inhibited by ChREBP down-regulation, although without statistical significance (Fig. 2C). These data suggested that glycolytic and lipogenic pathways were inhibited by ChREBP knockdown. Moreover, we found that the mRNA of p53 (p = 0.058) and protein level of phospho-p53 were increased (p = 0.002) following ChREBP knockdown. However, the mRNA levels of p21 decreased by about 20% and protein level of p21 decreased by 40% (Fig. 2D,B). These data indicated that the p53 pathway was activated but p21 was inhibited following ChREBP knockdown in HT29 cells.

**Figure 3:**
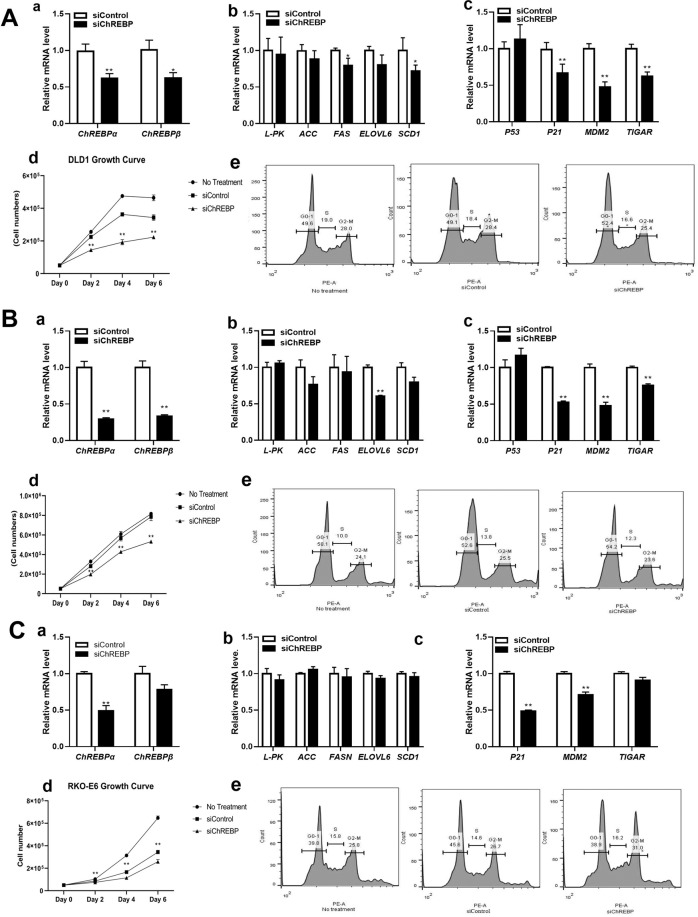
Effects of ChREBP knockdown on cell proliferation and cell cycle in DLD1, SW480 and RKO-E6 cell lines. (**a**) Relative mRNA levels of ChREBP α and ChREBP β after siRNA transfection for 48 hours in DLD1(A), SW480 (B) and RKO-E6 (C) cells. (b) Relative mRNA levels of glycolytic and lipogenetic genes after siRNA transfection for 48 hours in DLD1 (A), SW480 (B), and RKO-E6 (C) cells. (**c**) Relative mRNA levels of p53 related genes in in DLD1(A), SW480 (B) and RKO-E6 (C) cells. (**d**) Growth curve of DLD1(A), SW480 (B) and RKO-E6 (C) cells after the treatment of siRNA at different time points. (**e**) Cell cycle of DLD1(A), SW480 (B) and RKO-E6 (C) cells after siRNA transfection for 48 hours. n = 4. *p < 0.05; **p < 0.01.

ChREBP was also knocked-down in other colon cancer cell lines, including DLD1 (Fig. 3A), SW480 (Fig. 3B) and RK0-E6 (Fig. 3C). With the siChREBP transfection, ChREBP α/β mRNA levels were decreased by 40% and 70% in DLD1 and SW480 cells, respectively, when compared to cells treated with siControl (Fig. 3Aa and Ba). Lipogenic genes, FAS and SCD1 were significantly inhibited after siChREBP treatment in DLD1 cells (Fig. 3Ab). In SW480, ELOVL6 was significantly repressed (Fig. 3Bb) after ChREBP knockdown. TP53-inducible glycolysis and apoptosis regulator (TIGAR), a glycolysis regulator was decreased after siChREBP treatment in both DLD1and SW480 cells (Fig. 3Ac and Bc). These results indicated that ChREBP knockdown inhibited glycolysis and lipogenesis. Similar to HT29 cells, gene expression of p21 was decreased after ChREBP knockdown in both DLD1 (Fig. 3Ac) and SW480 cells (Fig. 3Bc). Mouse double minute 2 homolog (MDM2), a negative regulator of p53, decreased upon ChREBP downregulation in both DLD1 (Fig. 3Ac) and SW480 cells (Fig. 3Bc). 50% of ChREBPα was downregulated in a p53 function loss cell line RKO-E6 (Fig. 3Ca), but the glycolytic and lipogenic genes did not significantly alter (Fig. 3Cbc), indicating that ChREBP mediated glycolysis and lipogenesis was p53 dependent. Gene expression of p21 was still inhibited after ChREBP knockdown, consistent with all other cell lines (Fig. 3Cc).

### ChREBP knockdown inhibited colon cancer cell proliferation and induced cell cycle arrest

**Figure 4:**
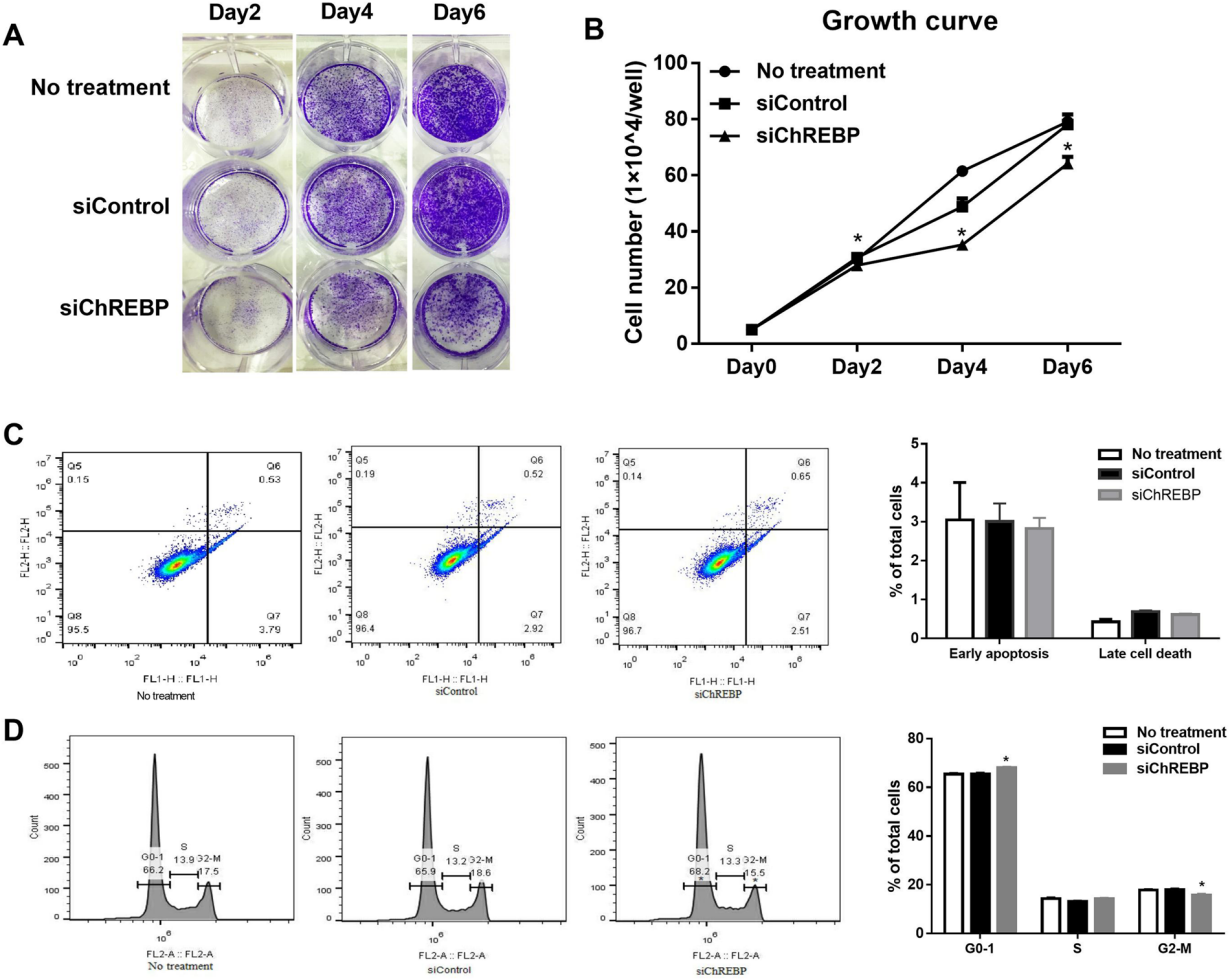
ChREBP knockdown inhibited HT29 proliferation and induced cell cycle arrest. (**A**) Crystal violate staining of HT29 cells after being transfected with or without siRNA for 2 days, 4 days and 6 days. No treatment, no siRNA was transfected in HT29 cells. siControl, non-targeting siRNA; siChREBP, siRNA targeting ChREBP. (**B**) Quantification of the crystal violate staining, indicating inhibited cell proliferation after being transfected with ChREBP siRNA for 2 days, 4 days and 6 days. (**C**) Cell apoptosis assessment with flow cytometry after treatment with or without siRNA for 48 hours, showing no changes after ChREBP knockdown. Lower right corner represents early apoptosis, while upper right corner represents late apoptosis or cell death. (**D**) Cell cycle measurement with flow cytometry after treatment with or without siRNA for 48 hours, showing cell cycle arrest after ChREBP inhibition. All assessments were repeated 3 times. n = 4. *p < 0.05.

After the transfection of siChREBP, cell proliferation measured with the staining of crystal violet was significantly inhibited at day 2, day 4 and day 6 post transfection when compared to cells treated with no siRNA or non-targeting siRNA in HT29 cells (Fig. 4A), DLD1 cells (Fig. S2Aa) and SW480 cells (Fig. S2Ba). After quantification of the crystal violet staining, HT29 cell numbers were reduced by 10%, 28% and 20% at day 2, day 4 and day 6 post transfection, respectively, when compared to the siControl group (Fig. 4B). In DLD1 cells, cell numbers were reduced by 14%, 31% and 25% on Day2, Day4 and Day6, respectively, following ChREBP knockdown when compared to the siControl group (Fig. 3Ad). In SW480 cells, after ChREBP knockdown, cell numbers were reduced by 30%, 25% and 32% on Day2, Day4 and Day6, respectively (Fig. 3Bd). In the p53 function loss cell line RKO-E6, cell proliferation was significantly inhibited as well (14%, 31%, 25% on Day2, Day 4 and Day 6, respectively) (Fig. 3Cd). These data indicated that ChREBP knockdown significantly inhibited cell proliferation in colon cancer cells, and seemed this inhibition was independent of p53. Cell apoptosis was measured at 48 hours after ChREBP siRNA transfection via flow cytometer, but showed no differences when compared to cells treated with no siRNA or non-targeting siRNA in HT29 cells (Fig. 4C), DLD1 cells (Fig. S2Ab) and SW480 (Fig. S2Bb). Cell cycle regulation genes measured after siRNA transfection for 48 hours showed that the ratio of cells in G0–1 phase increased significantly but the ratio of cells in G2-M phase was decreased significantly when compared to the siControl group in HT29 cells (Fig. 4D). However, the ratio of cells in DNA synthesis (S phase) did not change significantly after ChREBP knockdown (Fig. 4D). These data indicated that ChREBP inhibition induced cell cycle arrest in the G0–1 phase, and less cells went through mitosis when compared to the siControl group in HT29 cells. The gene expression of cell proliferation marker ki67 was significantly inhibited after ChREBP knockdown, consistent with the proliferation growth curve. When mRNA levels of cell cycle-relevant genes were examined, we found that cyclin B1 and cyclin E1 were significantly decreased with ChREBP down-regulation (Fig. 2E). This data suggested inhibition of cell proliferation. Cell cycles after ChREBP knockdown were also examined in other colon cancer cells (DLD1 and SW480). In DLD1, more cells in G0–1 phase but less cells in the phase of S were observed upon ChREBP downregulation (Fig. 3Ae). The same trend was also observed in SW480 although without significance (Fig. 3Be). However, this trend was disappeared and instead, less cells were in G0–1 but more cells were accumulated in G2-M (Fig. 3Ce). These data suggested that ChREBP inhibition arrested colon cancer cell cycle likely in a p53-dependent manner.

## Discussion

Growing evidence indicates that ChREBP plays an essential role in cancer development, growth and progression because of its strong ability of regulating glycolysis and lipogenesis. It has been reported that suppression of ChREBP mRNA inhibits cell proliferation *in vitro* and reduces tumor growth *in vivo*^26,32^. Moreover, ChREBP protein expression was reported to be positively correlated with tumor progression in breast cancer^21^ and hepatocellular carcinoma (HCC)^22^. However, the relationship between ChREBP expression and colon cancer malignancy has not been investigated. The reasoning to explore the ChREBP expression in colon cancer is that disturbed glucose and lipid metabolism patients show a higher prevalence of colon cancer^14,15^, and these metabolic pathways are strongly regulated by the transcription factor ChREBP. In this study, we found that ChREBP mRNA and protein expression increased significantly when compared to the healthy colon, and their expression were positively correlated with colon malignancy. In normal colon tissue, there was only mild or medium ChREBP expression, but in colon cancers, the expression was prominent and the late stage colon cancer cells showed stronger ChRBEP staining than that seen in early stage colon cancer cells. The protein levels of glucose transporter (GLUT1 and GLUT2) and mRNA levels of ChREBP regulated lipogenic gene (*ELOVL6* and *SCD1*) showed a similar expression pattern, indicating increased glycolysis and lipogenesis with the progress of colon cancer. The increased ChREBP expression and increased glycolysis and lipogenesis may contribute to cancer progression by providing energy supply and building blocks for cancer proliferation. Our data indicate the possibility that ChREBP expression level might be used to evaluate colon cancer behavior.

In this study, we also found that ChREBP knockdown inhibited cell proliferation and induced cell cycle arrest without changes in apoptosis in cultured colon cancer cells. Glycolytic and lipogenic pathways were inhibited after ChREBP knockdown. P53 tended to increase but p21 and MDM2 were decreased significantly following ChREBP knockdown in colon cancer cell lines. The decreased cell proliferation post ChREBP knockdown could be caused by the inhibited anabolic pathways, which provide energy and building blocks for cell proliferation. P21 and Cyclin E regulate the first checkpoint G1 in cell cycle and the deficiency of p21 in most human cancers makes them G1 checkpoint defective. Cancer cells with damaged DNA proliferate more by accelerated cell cycling^33,34^. In our study, the decreased p21 and Cyclin E expressions in HT29 indicate the promotion of cancer cells entering G1 phase, supported also by the higher ratio of cells in this phase. G2/M checkpoint regulation is the second checkpoint within the cell cycle. It provides an opportunity for DNA repair by increasing the time for DNA repairment and by transcriptionally inducing gene expression and stopping cells with damaged DNA from entering the M phase. This regulation is critical to prevent cells from going through malignant transformation^35^. In our study, we found that fewer cells were in G2-M phase after ChREBP knockdown. This may indicate more DNA damage by ChREBP knockdown than without knockdown. However, this hypothesis requires further verification. Together, these results showed an important role of ChREBP in cell proliferation via redirecting glucose metabolism from oxidative phosphorylation to glycolytic and lipogenic anabolic pathways, as reported in other studies^21,26^. However, we did not find that ChREBP knockdown influenced cell apoptosis significantly as shown in other reports^26,36^. Therefore, the impact of ChREBP to apoptosis might be cell type dependent. Our *in vitro* results further confirmed the importance of ChREBP in redirecting glucose to anabolic pathways and suppression of the p53 pathway.

Interestingly, in the disrupted p53 function colon cancer cell line RKO-E6, cell proliferation was still suppressed by ChREBP knockdown, indicating the independency of p53 in this inhibition. However, the inhibition of metabolic (glycolytic and lipogenic) genes and cell cycle arrest with siChREBP were abolished in RKO-E6 cells, indicating the dependency of p53 in energy metabolism and cell cycle.

Our findings obtained from colon cancer tissue and cultured cells indicate an essential role of ChREBP in cell proliferation and tumorigenesis. It is reported that ChREBP is needed to support maximal growth of hepatoblastoma induced by a mutant form of b-catenin^37^. Recently, it has been reported that ChREBP deletion strongly delayed or impaired hepatocarcinogenesis driven by AKT or AKT/c-Met overexpression in mice, further supporting a crucial role of ChREBP in tumorigenesis^38^. However, in the same report, the authors found that ChRBEP deletion had no impact on HCC development driven by co-expression of AKT and N-Ras protooncogenes^38^. Moreover, ChREBP down-regulation was found to play an essential role in mediating the transformation of epithelial to mesenchymal cells during non-small-cell lung carcinoma metastasis^39^. Therefore, how ChREBP influences tumorigenesis appears to be tissue and gene background dependent. There are many reports showing the importance of lipid metabolism in tumorigenesis. Inhibition of lipogenic pathways strongly impairs epithelial-mesenchymal transformation that promotes migration and invasion of colon cancer cells^11^. Therefore, inhibiting lipogenic pathways via targeting ChREBP might represent a new therapeutic opportunity for colon cancer treatment.

Taken together, the present study showed the essential role of ChREBP in cell proliferation and its expression level is positively associated with colon cancer malignancy. An evaluation of ChREBP expression may contribute to colon cancer diagnosis and may shed light on colon cancer treatment and improvement of patient survival.

## Materials and Methods

Human normal colon and carcinoma tissue microarray was purchased from a commercial source (BC051110c, Alenabio, Xi’an, China). The tissue array contains 108 cases of colon cancer (5, 67, 31 and 5 in clinical stages I, II, III and IV, respectively) and 12 cases of normal tissue. In addition, fifty one cases of frozen colon cancer tissue (8, 20, 19 and 4 in clinical stages I, II, III and IV, respectively) and 20 samples of adjacent normal tissue were collected from the Second Affiliated Hospital of Shantou University Medical College, Shantou, China. This study has been approved by Shantou University Medical College Ethic Committee. All methods were performed in accordance with the relevant guidelines and regulations. Informed consent was obtained from all participants and/or their legal guardians.

### Immunohistochemistry

Immunohistochemistry was carried out following an established protocol^40^. Briefly, colon tissue microarray or paraffin tissue sections were dewaxed, rehydrated through graded ethanol and incubated with 3% hydrogen peroxide for 30 min. Antigen retrieval was performed by heating the sections to 95 °C in 0.01 mol/l citrate buffer (pH6.0) for 15 min. Slides were then washed in PBS for 15 min and treated with 10% normal horse serum for 30 min and incubated with primary antibody at 4 °C overnight. The reaction products were detected with the 3-amino-9-ethylcarbozole (AEC) substrate-chromogen kit after incubating with the secondary antibody (Dako REAL EnVision Detection Kit (Dako, Carpinteria, CA)) for 30 min and washing in 0.1 M PBS at room temperature. Staining with AEC resulted in red signals. The primary rabbit-derived polyclonal antibody for anti-ChREBP (Novus biologicals, NB400-135, USA) was used at a 1:200 dilution. For negative controls, primary antibodies were replaced with PBS.

### Quantitive assessment of ChREBP staining

Quantification of ChREBP staining was carried out as described in a previous study^41^. Briefly, tissue microarray sample ‘spots’ were viewed and photos were taken at 200× magnification. The intensity of ChREBP positive signals were measured with Image J software (National Institutes of Health, Bethesda, Maryland) using the threshold tool.

### Cell culture and siRNA transfection

The human colon cancer cell line HT29 (ATCC, HTB-38™) and DLD1 (Cell Bank of the Chinese Academy of Science) were cultured with complete RPMI-1640 medium (Gibco, USA) supplemented with 10% fetal calf serum (FCS), 1% peninclin/streptomycin at 37 °C in humidified 5% CO2 atmosphere. SW480 and RKO-E6 (Cell Bank of the Chinese Academy of Science) were cultured with complete high glucose DMEM (Gibco, USA) supplemented with 10% fetal bovine serum (ExCellbio, China) at 37 °C in humidified 5% CO2 atmosphere. For cell apoptosis and cell cycle assessements, cells were plated in a 12-well plate at a density of 1×10^5^ cells per well for siRNA transfection and then transfected for 48 hours using Lipofectamine™ RNAiMAX (Invitrogen) according to the manufacturer’s protocol with 10pmol ChREBP siRNA or control siRNA per well in full growth medium. Non-specific siRNA (siControl) and siRNA oligonucleotides targeting ChREBP (siChREBP) were synthesized by GenePharma, China. The ChREBP small interfering RNA sequences were 5′-GCACCCUUGGCAAACCUUUUU-3′ and 5′-AAAGGUUUGCCAAGGGUGCUU-3′. Non-targeting siRNA sequences were 5′-UUCUCCGAACGUGUCACGUUU-3′ and 5′-ACGUGACACGUUCGGAGAAUU-3′.

### Cell proliferation

For cell proliferation analysis, colon cancer cells were plated at 5 × 10^4^ cells per well in 12-well plates and cultured for 24 hours. Cells were fixed with 1% glutaraldehyde at 2, 4, and 6 days after siControl or siChREBP transfection and stored at 4 °C until all samples were harvested. 0.1% crystal violet (Solarbio, China) was used to stain cells for 30 min and then was extracted with 10% acetic acid. The absorbance of each well at 490 nm was measured using an ELISA plate reader (Bio-Rad, USA). Quadruple wells were used for each experimental condition, and all the experiments were repeated at least three times.

### Cell apoptosis

For apoptosis assays, colon cancer cells were seeded at 1× 10^5^ per well in 12-well plates and cultured for 24 hours. On the second day, cells were transfected with siControl or siChREBP. After 48 hours, cells were harvested with trypsin and washed 2 times in ice-cold PBS, resuspended in 100 μl of binding buffer and incubated with Annexin V-FITC (4 A Biotech, China) for 5 min at 4 °C in the dark. After staining, the cells were incubated with propidium iodide for 5 min at 4 °C in the dark and then assayed with a flow cytometer (Beckman Coulter, Germany)^42^. Software FlowJo (Becton, Dickinson & Company, USA) was used to analyze cell apoptosis.

### Cell cycle

Cell cycle analysis was performed with a cell cycle kit (Shanghai BestBio, China) according to the manufacture’s instructions^43^. Briefly, colon cancer cells were seeded at 1× 10^5^ per well in 12-well plates and cultured for 24 hours. On the second day, cells were transfected with ChREBP or control siRNA. After additional 48 hours, cells were harvested with trypsin and washed 2 times in ice-cold PBS before being fixed with 70% ethanol at −20 °C for 1 hour. Cells were then washed 2 times again with cold PBS and incubated with 20 μl RNase A at 37 °C for 30 min, followed by incubation with 400 μl propidium iodide for 30 min at 4 °C in the dark and then assayed with a flow cytometer (Beckman Coulter, Germany). Four replicates in each group and same experiment has been repeated for 3 times. Software FlowJo was used to analyze cell cycle.

### Real-time PCR

Total RNA was isolated by TRI-Reagent (Sigma-Aldrich, USA) from human normal and cancer colon tissue and colon cancer cell lines according to the manufacturer’s protocol. cDNA was obtained with a reverse transcription kit (Life Technologies) using 1 μg of total RNA. qPCR reactions were performed using the SYBR Green PCR Master Mix Kit (Takara, Japan) and the ABI 7500 PCR system (Applied Biosystems). For the colon cancer cell lines, each group of cells treated with no siRNA, siControl or siChREBP was analyzed in tetraploid, and the data were quantified according to the 2^-ΔΔCt^ method. The mRNA expression levels were normalized to 18 S and β2 microglobulin (B2M) in frozen colon tissue and cultured cells, respectively. The sequences of primers and probes are listed in Supplementary Table 1.

### SDS-PAGE and Western blot

To analyze cytosolic proteins, cultured cells were harvested and washed twice with ice-cold PBS and lysed in RIPA lysis buffer (Millipore, USA) for 30 min on ice. Lysates were cleaned by centrifugation at 12,000 g for 15 min at 4 °C. The protein concentration in the cell lysate was measured with a BCA kit (Pierce Biotechnology, USA). Identical amounts of lysate proteins (30 μg/well) were separated with 10% SDS-polyacrylamide gel electrophoresis. Proteins were then transferred onto a polyvinylidene difluoride membrane and incubated in a blocking solution consisting of 5% powered milk in TBST (10 mmol/L Tris–HCl (pH 8.0), 150 mmol/L NaCl, and 0.1% Tween 20) for 1 h, followed by immunoblotting with the respective antibodies. Primary antibodies were goat anti-mouse G6pc (Dako, Carpinteria, CA), rabbit anti-mouse ChREBP (Novus, NB400-135), rabbit anti-mouse β-actin (Cell signaling, 4967 S), rabbit anti-human p21 (Abcam, EFR362), rabbit anti-human phospho-p53 (Cell signaling, 9284), and rabbit anti-human caspase3 (Cell signaling, 9662) at 4 °C overnight. Subsequently, the membranes were washed with TBST for 30 min at room temperature before incubation with secondary antibodies. Goat anti-rabbit IgG-HRP (Dako, Carpinteria, CA) and rabbit anti-goat IgG-HRP (Dako, Carpinteria, CA) were used as secondary antibodies. Immunoreactive proteins were detected with the Odyssey Infrared imager, according to the manufacturer’s protocol (Li-Cor Biosciences, NE). Densitometric analysis was performed using the software Odyssey. Full-length blots are included in the supplementary data (Figs. S3,  S4).

### Statistical analysis

Results are represented as means ± SEM. If data were normally distributed and had equal variance, Student’s t-test was used. If the data were not normally distributed, Mann-Whitney U test was used. The effect of colon malignancy on mRNA and protein expression was analyzed with one-way ANOVA. p<0.05 was considered statistically significant. All figures and statistics were made with Graphpad Prism 7 (CA, USA).

## Supplementary information

Supplementary materials.
