# Supplementary material for: Carbohydrate response element binding protein (ChREBP) correlates with colon cancer progression and contributes to cell proliferation
Source: Sci Rep. 2020 Mar 6;10:4233. doi: 10.1038/s41598-020-60903-9 (PMC7060312; doi:10.1038/s41598-020-60903-9)
Supplement: Supplementary file 1 — Supplementary materials. [file 41598_2020_60903_MOESM1_ESM.docx]

**Carbohydrate response element-binding protein (ChREBP) correlates with colon cancer progression and contributes to cell proliferation**

Running title: ChREBP correlates with colon cancer progression

Yu Lei^1,2#^, Shuling Zhou^1,2#^, Qiaoling Hu^1^, Xueling Chen^1^ and Jiang Gu ^1,2*^

Department of Pathology and Provincial Key Laboratory of Infectious Diseases and Immunopathology, Collaborative and Creative Center, Shantou University Medical College, Shantou 515041, Guangdong, China

2. Jinxin Research Institute for Reproductive Medicine and Genetics, Chengdu Jinjiang Hospital for Maternal and Child Health Care, 66 Jingxiu Road, Chengdu, 610066, China

^#^ These authors contributed equally to this work

* Corresponding author

**Materials and Methods**

**Semiquantitive assessment of ChREBP staining**

Semiquantitative scoring was carried out as described in a previous study ^[41](#_ENREF_41)^. Briefly, tissue microarray sample ‘spots’ were viewed at 200× magnification and an overall score was assigned according to intensity and area of positive immunostaining staining. Sample scores are as follows: 0, no red staining at all; 1, light pink staining in a minority of tissue; 2, pink staining in the majority of the tissue; 3, strong red staining in the majority of the tissue and 4,very strong dark-red staining in all the tissue. To reduce the variation of scoring, all slides, including microarray, were scored by two observers (Y. L. and Q. H.) independently.

**Table S1. Primers for human cells**

| **Gene name** | **Forward primer sequence (5'-3')** | **Reversed primer sequence (5'-3')** |
| --- | --- | --- |
| ChREBPα | AGTGCTTGAGCCTGGCCTAC | TTGTTCAGGCGGATCTTGTC |
| ChREBPβ | AGCGGATTCCAGGTGAGG | TTGTTCAGGCGGATCTTGTC |
| B2M | GATGAGTATGCCTGCCGTGT | TGCGGCATCTTCAAACCTCC |
| 18S | CGGCTACCACATCCAAGGA | CCAATTACAGGGCCTCGAAA |
| L-PK | TGTCTGTGCCACACAGATGCT | CATTGGCGACATCGCTTGTCT |
| FASN | CGCTCGGCATGGCTATCT | CTCGTTGAAGAACGCATCCA |
| ACC | TACAACGCAGGCATCAGAAG | TGTGCTGCAGGAAGATTGAC |
| ELOVL6 | CAGCCCCAATGAACATGTCA | ATACAGAGCAGAAAACAGGAAAGATTT |
| SCD1 | TTCCCGACGTGGCTTTTTCT | AGCCAGGTTTGTAGTACCTCC |
| p53 | TGAAGCTCCCAGAATGCCAG | GCTGCCCTGGTAGGTTTTCT |
| p21 | TCTAGGAGGGAGACACTGGC | TGTCTGACTCCTTGTTCCGC |
| Cyclin A2 | GGACCAGGAGAATATCAACCCG | AAGGGGTGCAACCCGTCTC |
| Cyclin B1 | TGGGTCGGCCTCTACCTTT | TGTTGCTCGACATCAACCTCT |
| Cyclin D1 | GCCACCGACTTTAAGTTTGCC | GCTCAGTCAGGGCATCACAA |
| Cyclin E1 | CAGGGAGCGGGATGCG | GGTCACGTTTGCCTTCCTCT |
| MDM2 | AGGAGATTTGTTTGGCGTGC | TGAGTCCGATGATTCCTGCTG |
| TIGAR | ACTCAAGACTTCGGGAAAGGA | CACGCATTTTCACCTGGTCC |


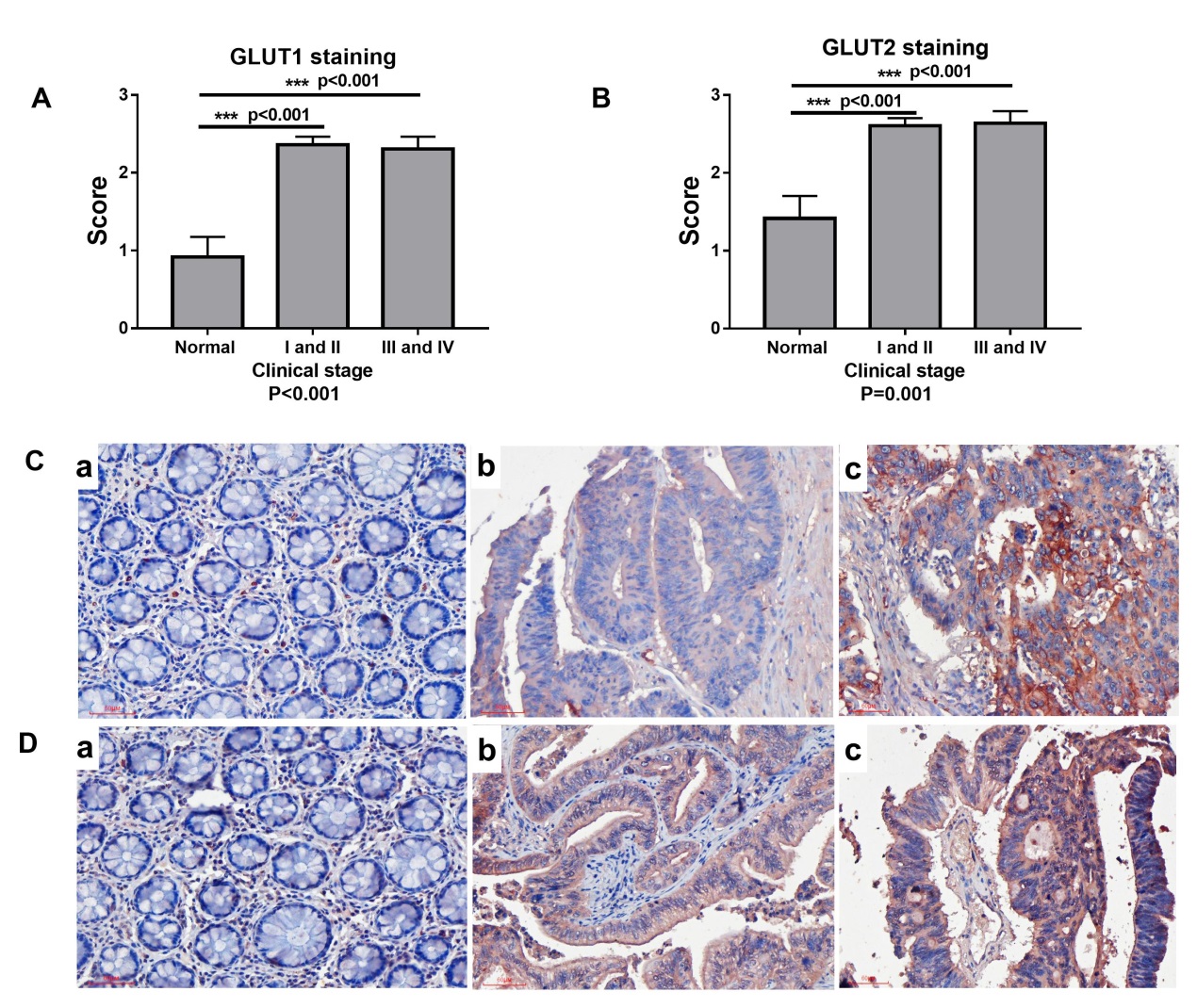


**Figure S1. GLUT1 and GLUT2 immunohistochemical stainings positively correlate to colon malignancy.** A, Histogram of scores of GLUT1 expression in different stages of colon tissue. B, Histogram of scores of GLUT2 expression in different stages of colon tissue. 0, absent of positive signal; 1, mild staining; 2, medium staining; 3, strong staining; 4, very strong staining. C, GLUT1 immunohistochemical positivity correlates with colon malignancy. D, GLUT2 immunohistochemical staining positivity correlates with colon malignancy. a, healthy colon tissue; b, clinical stages I and II colon cancer tissue; c, clinical stages III and IV colon cancer tissues. Photos were taken under the magnification of 200×. Scale bar=30 μm. ***P<0.001.


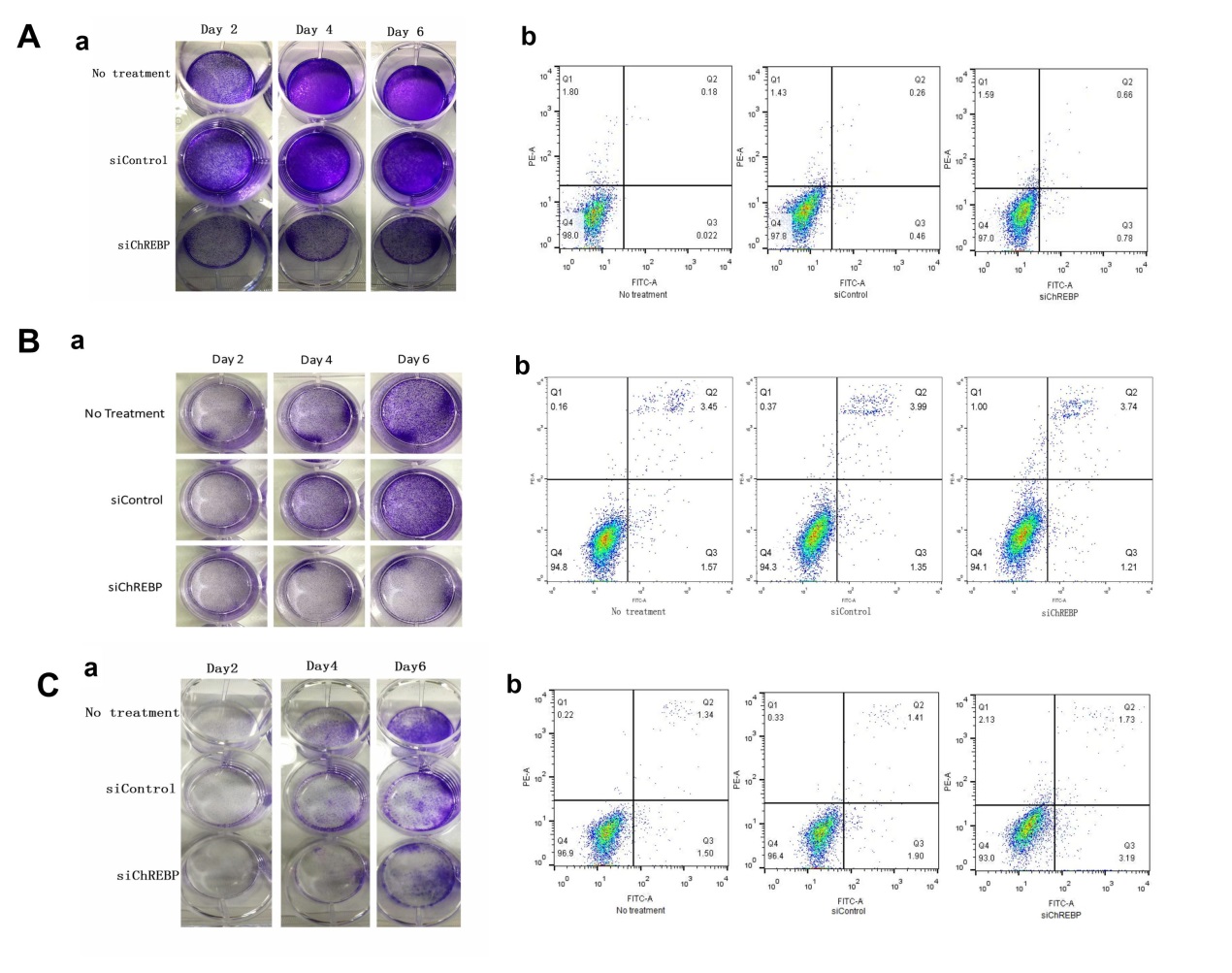


**Figure S2. ChREBP knockdown inhibited cell proliferatin of DLD1, SW480 and RKO-E6 cell lines without changing cell apoptosis.**  Crystal violate staining of DLD1 (Aa) SW480 (Ba) RKO-E6 (Ca) cells after being transfected with or without siRNA for 2 days, 4 days and 6 days. No treatment, no siRNA was transfected in HT29 cells. siControl, non-targeting siRNA; siChREBP, siRNA targeting ChREBP. Cell apoptosis assessment with flow cytometry after treatment with or without siRNA for 48 hours, showing no changes after ChREBP knockdown in DLD1 (Ab) SW480 (Bb) RKO-E6 (Cb) cells. Lower right corner represents early apoptosis, while upper right corner represents late apoptosis or cell death.


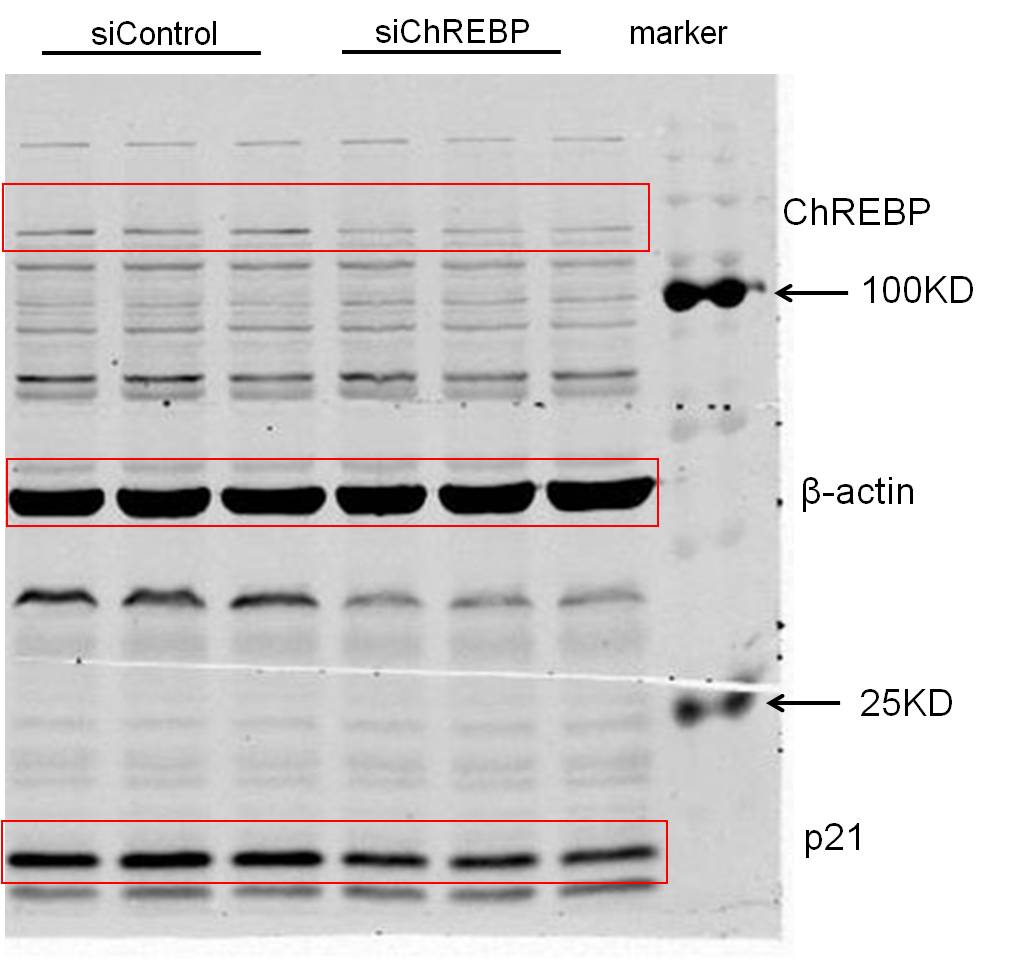


**Figure S3**. Full-length membrane used for quantification of ChREBP, p21 and β-actin. A full membrane was cut into 3 pieces and incubated with ChRBEP, β-actin and p21 antibody, respectively. Protein immunoreactive bands were visualized with the Odyssey Infrared imager (Li-Cor Biosciences, NE).


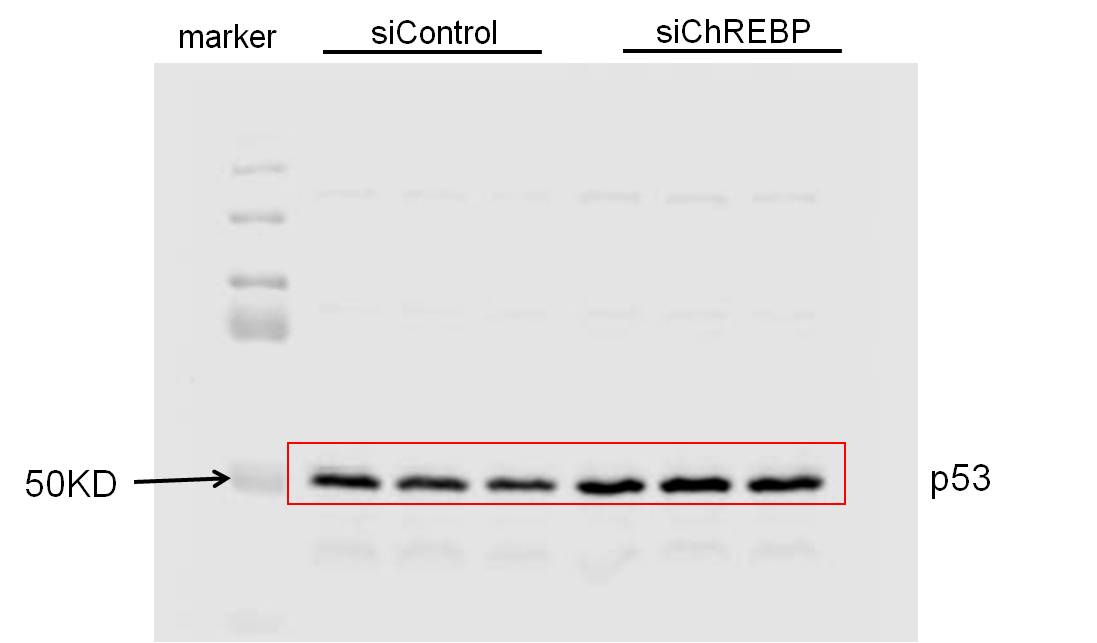


**Figure S4**. Full-length membrane used for quantification of phospho-p53. Protein immunoreactive bands were visualized with the Odyssey Infrared imager (Li-Cor Biosciences, NE).
